# Supplementary material for: Structural basis of HEAT‐kleisin interactions in the human condensin I subcomplex
Source: EMBO Rep. 2019 Mar 12;20(5):e47183. doi: 10.15252/embr.201847183 (PMC6501013; doi:10.15252/embr.201847183)
Supplement: Supplementary file 1 — Expanded View Figures PDF [file EMBR-20-e47183-s001.pdf]

Expanded View Figures

Figure EV1. Secondary structures and structure-based sequence alignment of human CAP-G and CAP-H.

A Secondary structures and structure-based sequence alignment of human CAP-G (hCAP-G), *Xenopus laevis* CAP-G (XCAP-G), and *Saccharomyces cerevisiae* YCG1. The secondary structural elements of hCAP-G and YCG1 are drawn above and below the sequence alignments, respectively. Identical and homologous residues are shown on black and gray backgrounds, respectively. The colored circles indicate residues of hCAP-G that interact with hCAP-H (green) or bind to HEPES (red). Residues of YCG1 that interact with BRN1 and dsDNA are labeled with light blue and red, respectively. The YC1 and YC2 regions indicate residues essential for DNA binding defined by Kschonsak *et al* [17].

B Structure-based sequence alignment of hCAP-H, XCAP-H, and BRN1. The secondary structural elements of hCAP-H and BRN1 are drawn above and below the sequence alignments, respectively. Identical and homologous residues are shown on black and gray backgrounds, respectively. The colored circles indicate residues of hCAP-H that interact with hCAP-G (orange) and residues of BRN1 that interact with YCG1 (purple) or dsDNA (red). BC1, BC2, latch, and buckle regions defined by Kschonsak *et al* [17], and motifs III and IV of CAP-H are also shown in Fig 1A.

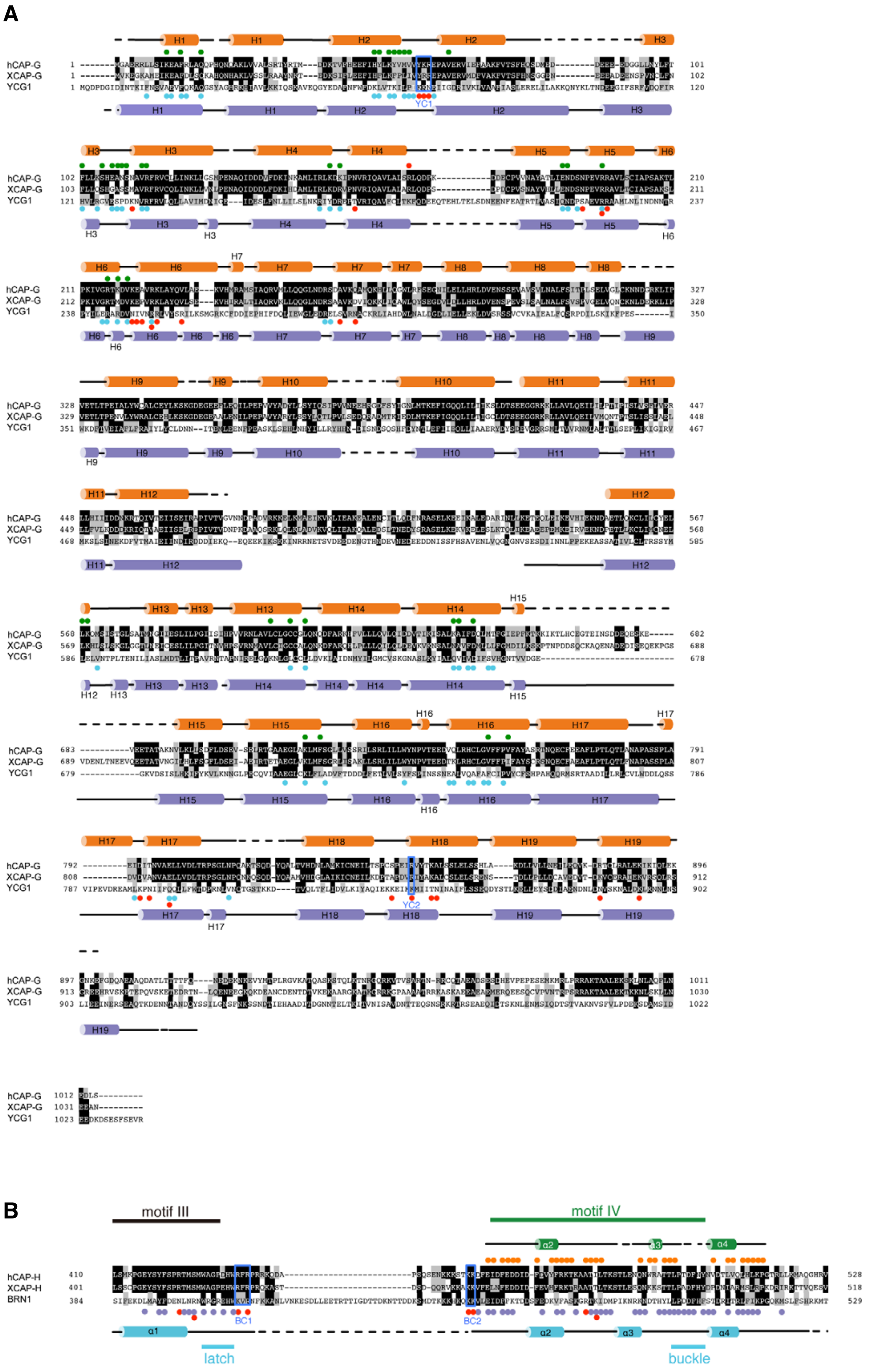

Figure EV1.

© 2019 The Authors

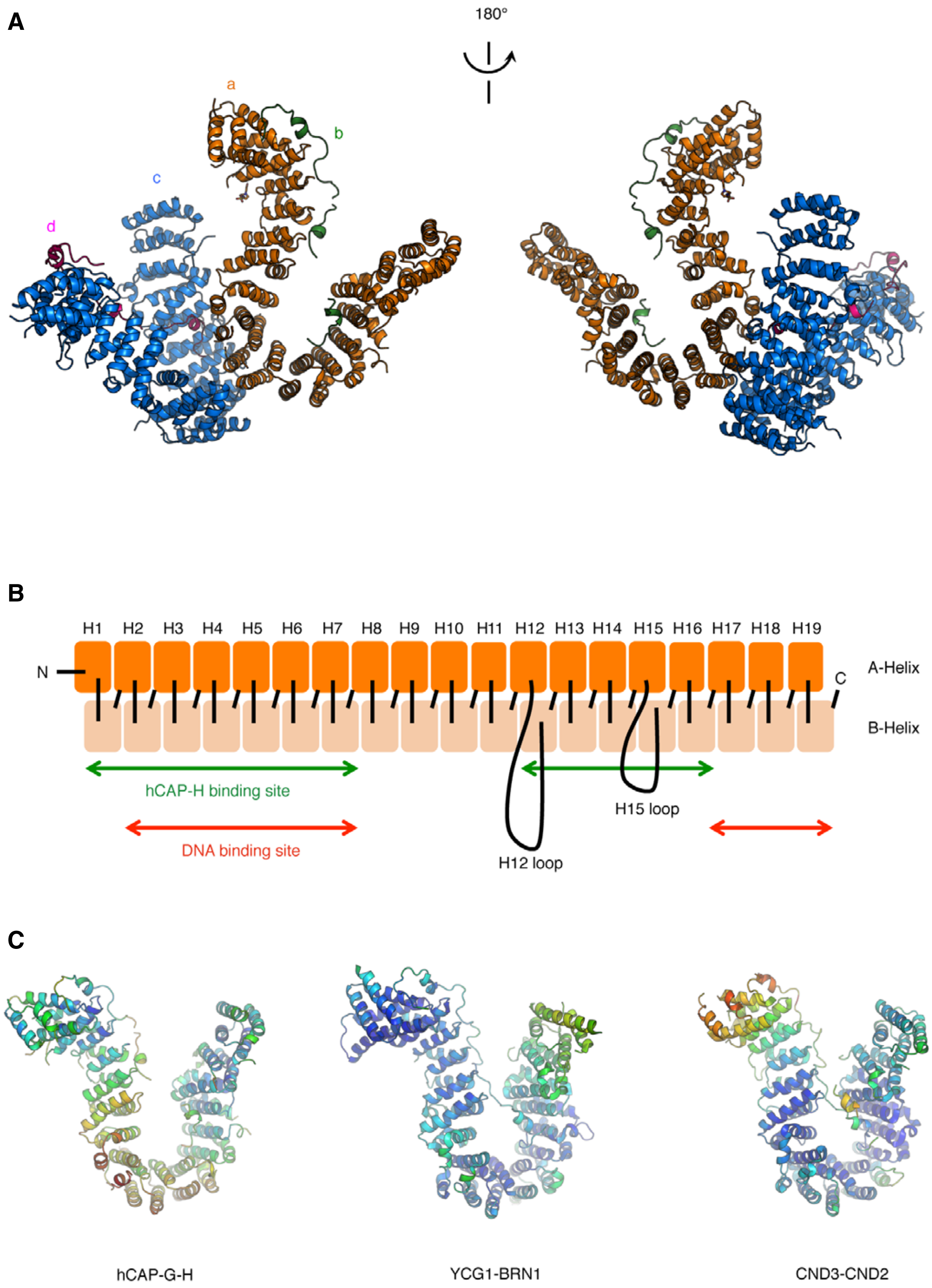

Figure EV2. Structure of the hCAP-G-H subcomplex.

A Two molecules of the hCAP-G-H subcomplex in the asymmetric unit, shown by orange (hCAP-G; a) and green (hCAP-H; b), and blue (hCAP-G; c) and pink (hCAP-H; d) ribbon representations. The pink stick model indicates HEPES. Note that HEPES bound only one of the two hCAP-G molecules (a-molecule) present in the asymmetric unit.

B Schematic illustration of the structure and domain organization of hCAP-G. Two antiparallel helices (A and B helices) comprising each HEAT repeat are colored in orange and light orange, respectively. The binding sites of hCAP-H and DNA are indicated by the green and red double-headed arrows, respectively. The H12 loop (residues 479–553) connecting the H12A and H12B helices, and the H15 loop (residues 660–690) connecting the H15A and H15B helices are shown by black loops.

C Comparison of the b-factors of the hCAP-G-H subcomplex with its related structures. The structures of hCAP-G-H (left), *S. cerevisiae* YCG1-BRN1 (PDB ID: 5OQQ; middle), and *S. pombe* CND3-CND2 (PDB ID: 5OQR; right) are shown as a ribbon model colored by b-factor. The b-factors are shown in warm (high b-factors) to cool colors (low b-factors).

© 2019 The Authors

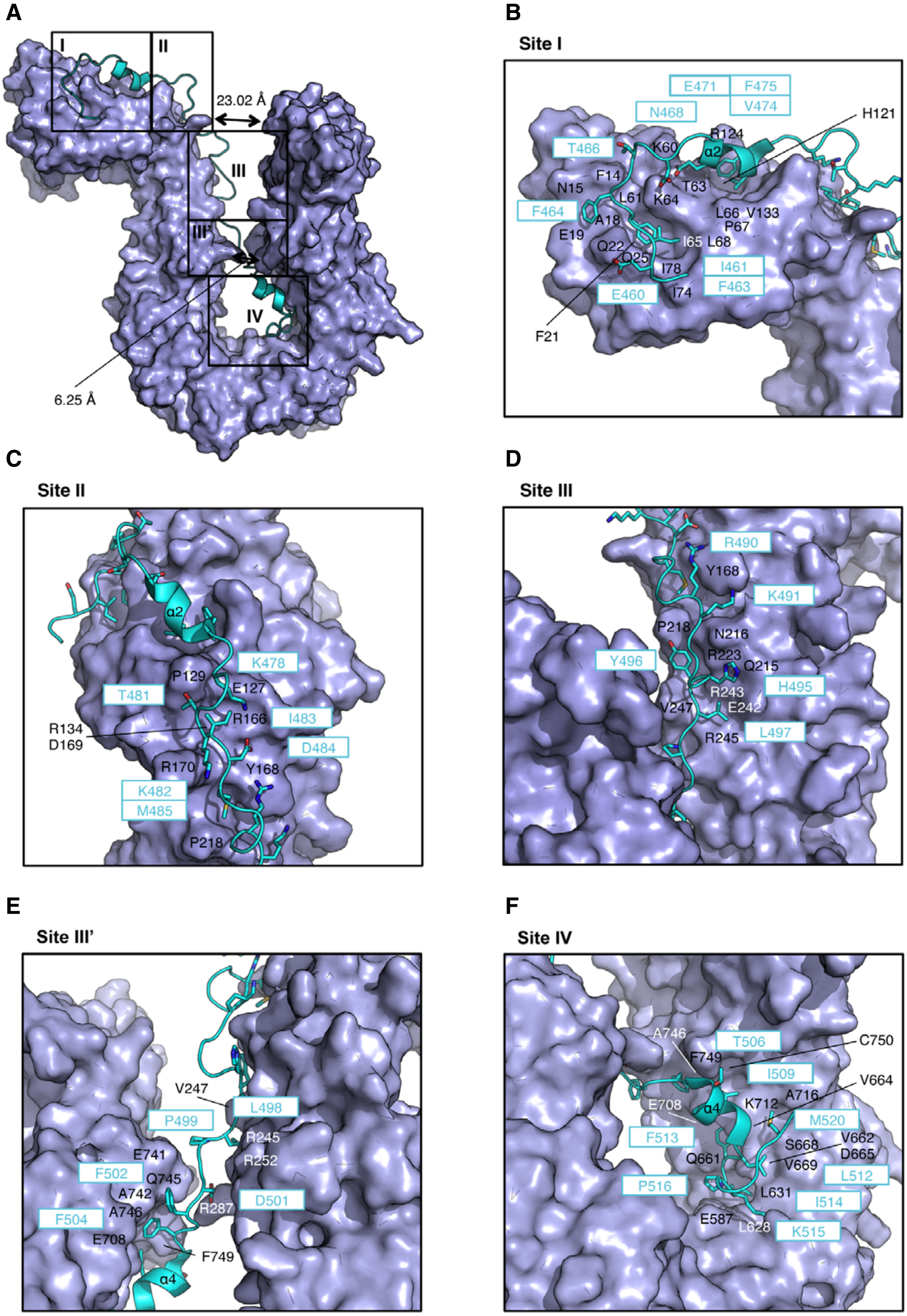

Figure EV3. Structural details of the interaction between YCG1 and BRN1.

A The molecular surface of YCG1 is shown in purple. BRN1 is shown as a light blue ribbon model. The five major contact sites (I, II, III, III′, and IV) are boxed. The representative structure was generated to use the a- and c-molecules from the reported structure of the YCG1-BRN1 subcomplex (PDB ID: 5OQQ).

B–F Zoomed-in views of sites I–IV. Residues of YCG1 and BRN1 are labeled in white or black and light blue, respectively.

 © 2019 The Authors

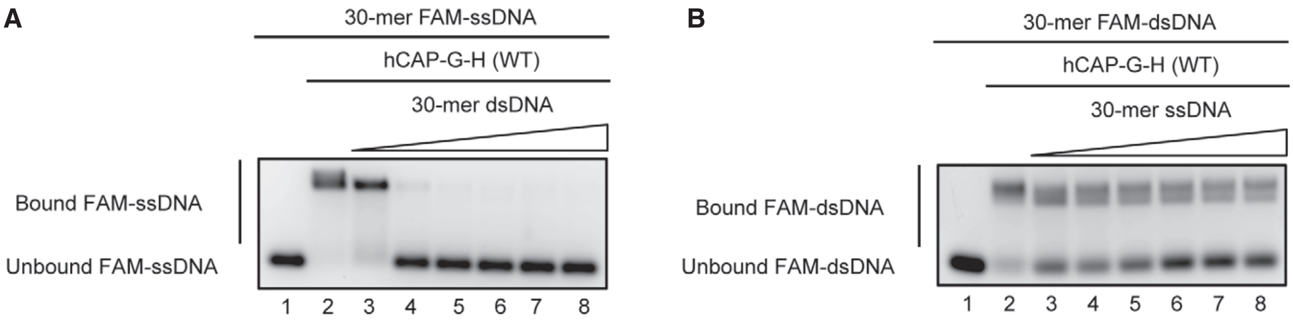

Figure EV4. Competition between double-stranded DNA (dsDNA) and single-stranded DNA (ssDNA) for hCAP-G-H binding.

A 30-mer FAM-ssDNA was incubated with no protein (lanes 1), WT hCAP-G-H (WT; lanes 2–8), or increasing amounts of 30-bp dsDNA (WT; lanes 2–8).

B 30-bp FAM-dsDNA was incubated with no protein (lanes 1), WT hCAP-G-H (WT; lanes 2–8), or increasing amounts of 30-mer ssDNA (WT; lanes 2–8).

© 2019 The Authors
